# Micronutrient deficiencies and cardiac health

**DOI:** 10.3389/fnut.2022.1010737

**Published:** 2022-10-14

**Authors:** Shazia Rehman, Zhang Jianglin

**Affiliations:** ^1^Department of Biomedical Sciences, Pak-Austria Fachhochschule, Institute of Applied Sciences and Technology, Haripur, Pakistan; ^2^Department of Dermatology, Shenzhen People's Hospital, The Second Clinical Medical College, Jinan University, The First Affiliated Hospital, Southern University of Science and Technology, Shenzhen, China; ^3^Candidate Branch of National Clinical Research Center for Skin Diseases, Shenzhen, China

**Keywords:** dietary habits, cardiac disease prevention, IHD, stroke, Spain, dietary and nutritional patterns, cardiovascular disease

## Abstract

Inadequate diet and nutritional quality are potentially correlated with an escalated risk of cardiac-related morbidity and mortality. A plethora of knowledge is available regarding the influence of heart-healthy dietary patterns in response to disability-adjusted life years (DALYs), yet little is known regarding the best approaches to adopt. In response, the present investigation aims to bridge this knowledge gap by implementing mathematical machine learning grey methodology to assess the degree of influence and the potential contributing factors in DALYs due to ischemic heart disease and stroke, in conjunction with the Hurwicz (Min-Max) criterion. The outcomes highlighted that a diet low in fruits is a potential contributor to IHD-related DALYS, whereas a diet low in vegetables is a more grounded contributor to stroke-related DALYs in Spain, among others. Moreover, the Hurwicz approach highlighted IHD to be more impacted due to dietary and nutritional factors than stroke. In conclusion, our investigation strongly supports a balanced diet and precision nutrition guidelines as a strategy for reducing cardiac-related diseases in the Spanish population. It is a public health primary consideration to build an ambiance that encourages, rather than hinders, compliance with cardioprotective dietary practices among all people.

## Introduction

Cardiovascular disease (CVD) continues to be the major cause of mortality and disability in industrialized nations, although its worldwide burden has progressively declined over recent years (1). In actuality, one in three fatalities in the United States (US) and one in every four in Europe are caused by CVD (2, 3). Among the non-communicable diseases, CVDs are the leading driver of deaths in Spain and account for ~33% of the total mortality. In this demographic, ischemic heart disease is the primary cause in men whereas cerebrovascular disease (stroke) is the main cause of death in women (4–7). In addition, cardiac incidence has sharply increased in developing economies over the past 25 years, becoming the second leading cause of years lost from premature death in the majority of these nations (8, 9), partly as a result of their adoption of Western dietary habits. Despite advancements in treatment, the substantial global burden of CVDs highlights the need for efficient preventative and therapeutic measures to slow the progression of this prevalent chronic disease. The dietary risk appears to be a key focus for cardiac prevention and therapy since it accounts for ~one-third of worldwide mortality (10).

Considerable evidence claims to support the cardiac advantages of healthy eating practices. Nutrition and dietary behaviors are critical to maintaining cardiac health. They are also essential in the prevention and management of CVDs and chronic illnesses. Globally, dietary variables were responsible for about 11 million fatalities and ~255 million disability-adjusted life years (DALYs) during the year 2017 (11). Dietary behaviors include the balance, diversity, and mixture of foods and drinks ingested regularly. This covers all foods and fluids, whether they are cooked or eaten at home or elsewhere. Adherence to heart-healthy eating behaviors is strongly linked to potential cardioprotective effects (12). Since CVD develops throughout neonatal development and premature infancy, it is imperative to develop cardioprotective eating behaviors early in our lifespan, particularly preconception, as well as to sustain them throughout one's life (13). Food-based dietary behavior assistance is intended to promote nutrient sufficiency, heart health, and overall well-being while taking into account personal preferences, cultural and religious customs, and life phases. Cardioprotective dietary habits, or those linked to lower risk of cardiac disorders, usually focus on fruit, and vegetable intake, whole-grain meals, lean protein resources, vegetable oils, and negligibly processed meals. Additionally, these behaviors include a minimal intake of salty and sugary beverages and meals.

Multiple studies integrate healthy eating practices to lower plasmatic levels of pro-inflammatory indicators (14), whereas a Western-style diet (a diet heavy in protein) is linked to greater levels of low-grade inflammatory activation (15). Therefore, a healthy diet is recommended by cardiovascular guidelines. A better blending of various meals and minerals is possible with dietary intervention. Due to the obvious synergistic health impacts among them, healthy eating habits ultimately promote a stronger amplitude of positive benefits than the prospective benefits of nutrient supplementation. As per the existing scientific literature, healthy eating habits are characterized by a high intake of fiber, vitamins, antioxidants, minerals, monounsaturated fatty acids/polyunsaturated fatty acids, low intakes of sodium chloride, refined sugar, saturated and trans fats, polyphenols, and high intakes of complex carbs with minimal glycemic loads (16). This equates to significant consumption of fresh produce, such as fruits, vegetables, lentils, fish, and shellfish, as well as nuts, seeds, whole grains, vegetable oils, primarily extra virgin olive oil, including dairy products, while consuming less sugary snacks, red and processed meat, and carbonated drinks. For Cardiac outcomes, the Mediterranean and DASH diets have received considerable attention. The descending of reduced inflammation and improved body weight management, which both enhance other potential parameters and are associated with fewer clinical occurrences, may both lessen the prevalence of CVD (16, 17).

The significance of dietary behaviors concerning public healthcare has spurred consumer interest in food and nutraceutical constituents, particularly fruits and vegetables. The American Heart Association (AHA) Nutrition Committee and the European Society of Cardiology (ESC) potentially recommend the regular intake of both fruits and vegetables in multiple servings to reduce cardiovascular risk (18, 19). These guidelines are mostly founded on epidemiological research and meta-analyses (19–22). A meta-analysis of 71 clinical and 12 epidemiological investigations revealed a significant inverse relationship between vegetable and fruit consumption (with CRP and TNF levels) and a direct relationship with an elevated accretion of T cells (22). Likewise, in another cohort investigation of 792 subjects (age 70 years), the interconnections between particular single meals (inclusive of fruits and vegetables) and indicators of systemic inflammation were found. According to the investigators, consuming more fresh fruits was potentially proven to reduce CRP levels. No substantial linkage was revealed against vegetables. Comparable findings were observed between fibrinogen levels and fruit intake independently or in conjunction with vegetables (23).

Multiple objectives are a typical challenge with systemic issues, which makes decisions more ambiguous. Discovering methodologies that incorporate the strongest criteria in the decision-making procedure that directly affect decisions is essential in this context to reduce inaccuracies (24–26). However, usually, this technique is challenging to implement since the decision-making criteria parameters fluctuate elevating the level of ambiguity in the final response. In the sphere of health, these protocols are far more challenging since they encompass not only technological issues as well as the human aspect, which generates conflicts of interest and impedes the ultimate decision. In consequence, multi-criteria decision analysis (MCDA) has been employed in its various forms to strengthen healthcare structures overall (27–29). The association between cardiac-related prevalence and related dietary and nutritional variables has been explored in these researches *via* a variety of methodologies. The greatest ways to improve the caliber of research work and its application are, however, scarcely understood.

In response to the above-stated research query, we integrated the grey machine learning approach with Hurwicz's approach to seek a comprehensive understanding of dietary and nutritional patterns on ischemic heart disease (IHD) and stroke prevalence in the Spanish population. Therefore, by targeting a broad variety of dietary and nutritional factors together with the prevalence of major cardiac-related disorders, we may be able to construct a more comprehensive and potential continuum of correlations. Ultimately, our work seeks to explore the missing gaps in the existing body of literature by measuring the nexus between vitamin A deficiency, a diet low in vegetables, a diet high in sodium, zinc deficiency, iron deficiency, and a diet low in fruits, with disability-adjusted life years (DALYs) of ischemic heart disease (IHD) and stroke. We implemented mathematical machine learning grey modeling of grey system theory to investigate potential interactions, these include Deng GRA, absolute GRA, and the second synthetic GRA. The purpose of the absolute GRA model is to provide integral closeness or proximity between two variables whereas Deng's GRA model just reveals partial closeness or proximity. The second synthetic GRA model, on the flip side, incorporates the characteristics of both Deng's GRA and absolute GRA models to offer a more comprehensive closeness also known as inclusive proximity. This closeness or proximity is also known as correlation in the current literature. The core idea behind GRA models is to evaluate the degree of relationships between the study variables based on how comparable their geometric curves are to one another. To put it simply, the fundamental goal of GRA models is to quantify the proximity or closeness of two data sequences that indicate two curves from a distinct perspective. These insights set one GRA model apart from another. Dependent on the notion of an association between the reference series and the comparability series of the data, GRA can ascertain the potential factors of the given inputs. These models have higher precision and could produce more reliable solutions though with limited data.

Also, Hurwicz's (Min-Max) criterion was applied to perform a comparative evaluation of all the chosen dietary and nutritional variables with IHD and stroke DALYs within the Spanish population to ascertain which dietary and nutritional variable is potentially contributing to DALYs of IHD and stroke.

## Materials and methods

### Data source

All of the data analyzed in the current analysis is derived from the Global Burden of Disease (GBD 2019) study. DALYs were chosen as the unit of measurement for IHD and stroke, and dietary and nutritional components (vitamin A deficiency, a diet low in vegetables, a diet high in sodium, zinc deficiency, iron deficiency, and a diet low in fruits) were also chosen as DALYs. The collected data were for both sexes (male and female) and were expressed as percentages of the total population. All data were normalized as the first step before proceeding with the analysis.

### Grey analyses

#### Grey relational analyses (GRA)

GRA methods are one of the fundamental aspects of grey system theory, which was presented in 1982 by Chinese scholar Deng Julong to deal with ambiguous processes with partial information (30). The basic principle behind grey modeling is that the measure of closeness (or correlation) of the multilateral pattern of a given dataset representing the structural properties, could be adapted to foresee the closeness of a linkage among variables within the systems. A detailed description of the GRA method can be found in (31). The incorporation and effective application of grey approaches have been demonstrated in the literature in several scientific disciplines (32–36). The present investigation is carried out by engaging GRA methodologies. The GRA models are designed using SAS (2019), whereas Microsoft Excel software (2019) was used to solve the Hurwicz (Min-Max) criterion. The proposed analyses and modeling approaches are used in the paper to measure the level of influence and linkage between dietary and nutritional variables and IHD and stroke DALYs in a multifaceted way. The accompanying section summarizes the computing algorithms for grey relational models.

### Deng's grey relational analysis (GRA) model

Let *𝕐*_*i*_ be the reference series of the given data set denoting a dependent parameter and *𝕐*_*j*_ be the comparative sequences of the given data set denoting an independent parameter, then, the grey relational gradient (GRG), (the output of the GRA model) is depicted as γ_*ij*_ *or γ*(*𝕐*_*i*_, *𝕐*_*j*_) and can be accompanied by Rehman and Rehman (37)and Xuerui et al. (38):


$$\begin{array}{l} \gamma \left({𝕐}_{i}\;,\;{𝕐}_{j}\right)=\;\frac{1}{\hbar }\overset{\hbar }{\underset{𝓁=1}{\sum }}\gamma \left(Y_{i\left(𝓁\right)},Y_{j\left(𝓁\right)}\right) \end{array}$$

Where,


$$\gamma \left(Y_{i\left(𝓁\right)},Y_{j\left(𝓁\right)}\right)=\;\frac{\min_{k}\min_{𝓁}\left|Y_{i\left(𝓁\right)}-Y_{j\left(𝓁\right)}\right|+\zeta \;\max_{k}\max_{𝓁}\left|Y_{i\left(𝓁\right)}-Y_{j\left(𝓁\right)}\right|}{\left|Y_{i\left(𝓁\right)}-Y_{j\left(𝓁\right)}\right|+\;\zeta \;\max_{k}\max_{𝓁}\left|Y_{i\left(𝓁\right)}-Y_{j\left(𝓁\right)}\right|}$$

In this case, ζ* ϵ* (0, 1) denotes the distinguishing coefficient, and is generally assumed to be at 0.5.

### Absolute grey relational analysis (GRA) model

If *𝕐*_*i*_(*dependent*) *and 𝕐*_*j*_(*independent*) are two distinct data series connected within a system, so the algorithm for computing the absolute GRA model is shown below (31).


$$\begin{array}{l} \epsilon_{ij}=\frac{1+|{𝔯}_{i}|+|{𝔯}_{j}|}{1+|{𝔯}_{i}|+|{𝔯}_{j}|+|{𝔯}_{i}-𝔯|}, \end{array}$$

Where,


$$\begin{array}{l} {𝔯}_{i}=\;\overset{\hbar }{\underset{1}{\int }}{𝕐}_{i}^{0}dt,\;{𝔯}_{j}=\overset{\hbar }{\underset{1}{\int }}{𝕐}_{j}^{0}dt,\;{𝔯}_{i}{-\;𝔯}_{j}=\;\overset{\hbar }{\underset{1}{\int }}\left({𝕐}_{i}^{0}{-\;𝕐}_{j}^{0}\right)\;dt \end{array}$$

### Second synthetic grey relational analysis (SSGRA) model

The SSGRA model can be produced by incorporating the given equation (39).


$$\begin{array}{l} p=\vartheta \;\epsilon_{ij}+\left(1-\;\vartheta \right)\;\gamma_{ij}\vartheta \;\epsilon \;\left[0,1\;\right] \end{array}$$

**Figure 1:**
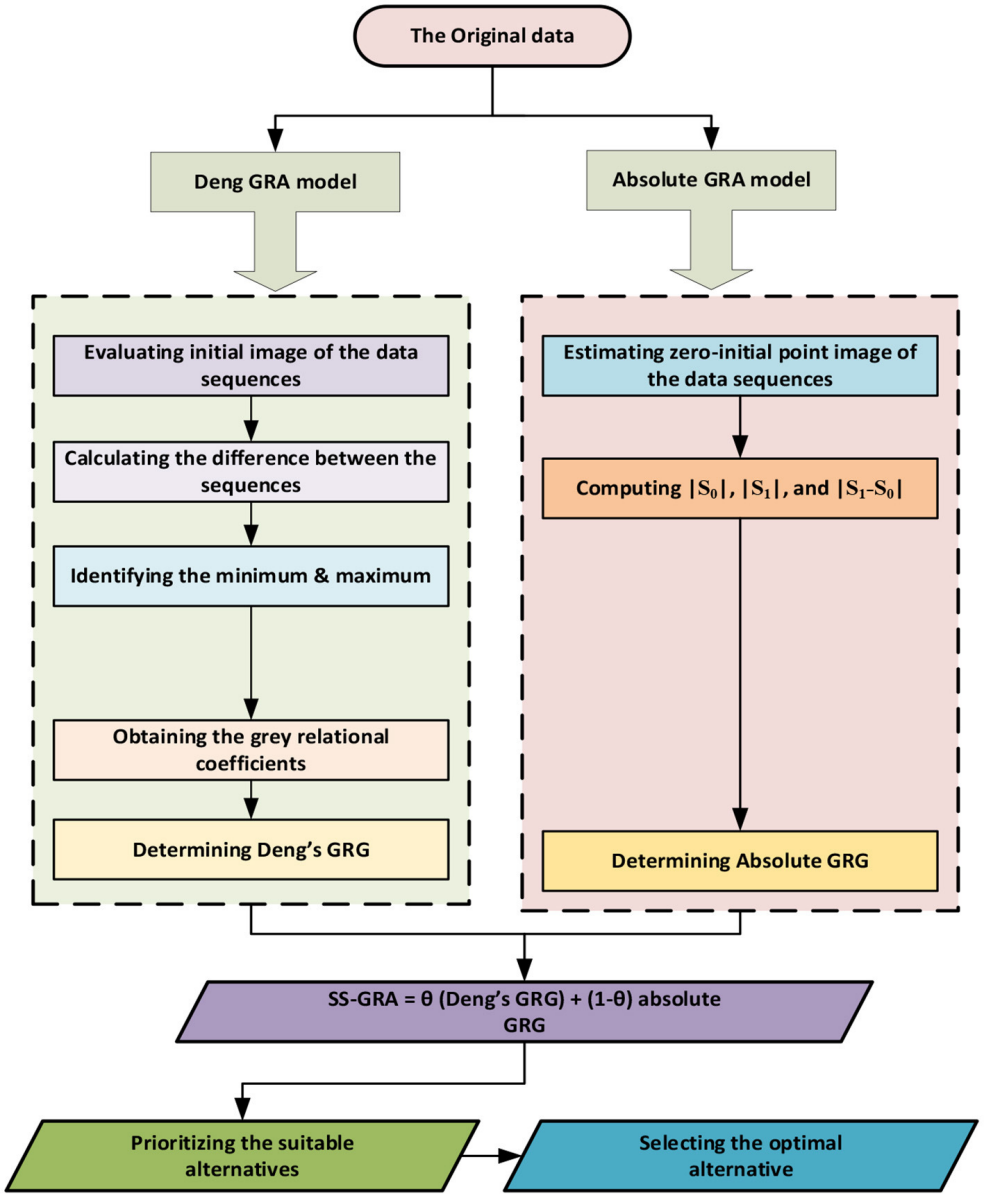
The methodological roadmap of grey modeling.

Where, ′p′ indicates the SSGRA model, ${}^{\prime }\epsilon_{ij}^{\prime }$ indicates the absolute GRA model whereas ′γ′ indicates the Deng GRA model outcomes across the two grey data series i.e., Y_i_ and Y_j_. Whenever a decision-maker seeks an inclusive evaluation that combines the pros of both ′ϵ′ and ′γ′ without endorsing one over another and so sets ϑ at 0.5. We assumed ϑ* at* 0.5 for our analysis. The detailed literature on grey modeling can be obtained from (31, 40). A graphical roadmap for the grey framework employed in our research is displayed in Figure 1.

## Results

**Figure 2:**
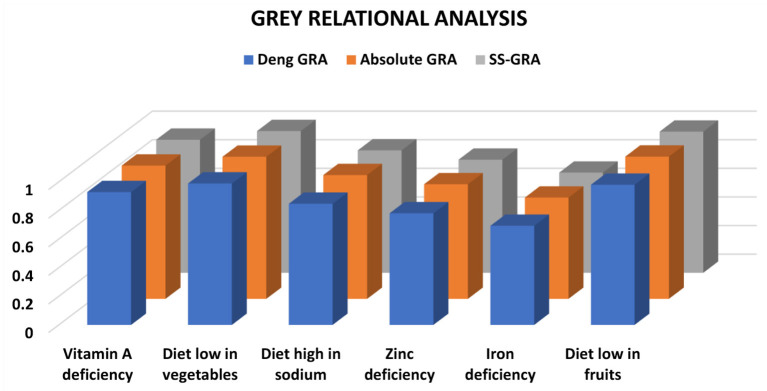
GRA assessment between IHD and nutritional variables.

**Figure 3:**
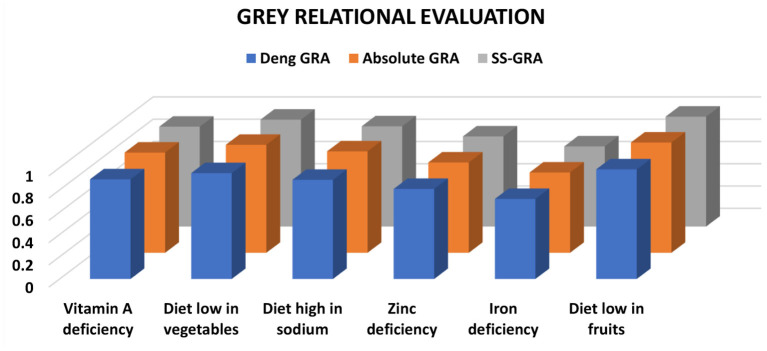
GRA assessment between stroke and nutritional variables.

The estimated outcomes for IHD based on the three grey relational models are shown in Table 1. A Diet low in vegetables (Deng GRA:0.9889, Absolut GRA: 0.9935, SS-GRA:0.9912) is shown to be highly associated with the number of DALYs attributable to IHD against Deng, absolute GRA, and SS-GRA models and ranked top as compared to the rest of the chosen variables. In addition, the variable diet low in fruits also appeared to be significantly associated with a very slight difference in strength. These data imply that a diet poor in vegetables and fruits has a significant impact on the occurrence of IHD. Furthermore, in all three grey relational models, the variables zinc (absolute GRA: 0.8014, Deng GRA:0.7806, SS-GRA:0.7910) and iron deficiency (Deng GRA:0.6931, absolute GRA:0.7083, SS-GRA:0.7007) had less effect on morbidity from IHD. A low-vegetable-and-fruit diet has been demonstrated to be a significant predictive risk factor for the higher incidence of IHD in the Spanish population. In Spain, the prevalence of IHD is considerably high and rising, but the control rate is lower than the needed threshold. These findings are consistent with previous epidemiological studies conducted in Spain, which discovered that a diet poor in vegetables is significantly associated with a frequent incidence of IHD (41–43). The graphical relationship between the cardiac DALYs and nutritional factors can be seen in Figures 2, 3.

**Table 1:** Grey relational assessment for IHD morbidity with nutritional risk factors.

	**Deng GRA**	**Absolute GRA**	**SS-GRA**	***r***
Vitamin A deficiency	0.9287	0.9321	0.9304	7.62
Diet low in vegetables	0.9889	0.9935	0.9912	18.04
Diet high in sodium	0.8472	0.8658	0.8565	6.50
Zinc deficiency	0.7806	0.8014	0.7910	3.11
Iron deficiency	0.6931	0.7083	0.7007	2.49
Diet low in fruits	0.9799	0.9947	0.9873	17.01
Ranking sequence based on GRA	Diet low in vegetables > Diet low in fruits > Vitamin A deficiency > Diet high in sodium > Zinc deficiency > Iron deficiency			
Ranking based on correlation	Diet low in vegetables > Diet low in fruits > Vitamin A deficiency > Diet high in sodium > Zinc deficiency > Iron deficiency			

r, Pearson's correlation coefficient, GRA, grey relational analysis, SS-GRA, the second synthetic grey relational analysis.

The findings in the case of stroke demonstrated that all risk variables maintained their positions against all grey relational models (Table 2). The risk factor diet low in fruits (Deng GRA:0.9848, absolute GRA:0.9904, SS-GRA:0.9876) ranked top while a diet low in vegetables (Deng GRA:0.9509, absolute GRA:0.9701, SS-GRA:0.9605) ranked second. The degree of association for the risk factor diet low in fruits was stronger than the rest of the risk factors. Considering the effect of zinc (Deng GRA:0.8090, absolute GRA:0.8112, SS-GRA:0.8101) and iron deficiency (Deng GRA:0.7192, absolute GRA:0.7212, SS-GRA:0.7202) on prevalence from stroke, the estimates against all grey relational models are found to show a comparatively frail linkage when compared with the rest of the risk factors. However, as compared to vitamin A deficiency, a diet high in sodium, zinc, and iron deficiency, a diet low in nutrients (vegetables and fruits) depicted a potential impact on increased DALYs from stroke as it holds 1st place throughout the analyses. The substantial associations between a diet low in fruits with stroke morbidity illustrated that it may be regarded as the strongest predictive risk factor to foresee DALYs and ultimately fatality from a stroke in the Spanish population. An abundance of evidence has shown stroke is the second leading cause of death and a top leading cause of disability in the Spanish population, with a stroke incidence of 187 cases per 100,000 persons per year (44). Our study's three grey relationship conclusions are congruent with a plethora of other stroke-related dietary and nutritional risk variables in Spain, where the incidence of stroke is growing and primary and secondary prevention should likely be fundamental health policy goals soon (45–47).

**Table 2:** Grey relational assessment for stroke morbidity with nutritional risk factors.

	**Deng GRA**	**Absolute GRA**	**SS-GRA**	**r**
Vitamin A deficiency	0.8951	0.9001	0.8976	7.01
Diet low in vegetables	0.9509	0.9701	0.9605	23.00
Diet high in sodium	0.8908	0.9110	0.9009	10.65
Zinc deficiency	0.8090	0.8112	0.8101	4.21
Iron deficiency	0.7192	0.7212	0.7202	1.22
Diet low in fruits	0.9848	0.9904	0.9876	23.68
Ranking sequence based on GRA	Diet low in fruits > Diet low in vegetables > Diet high in sodium > Vitamin A deficiency > Zinc deficiency > Iron deficiency			
Ranking based on correlation	Diet low in fruits > Diet low in vegetables > Diet high in sodium > Vitamin A deficiency > Zinc deficiency > Iron deficiency			

r, Pearson's correlation coefficient, GRA, grey relational analysis, SS-GRA, the second synthetic grey relational analysis.

We also employed Pearson's correlation coefficient to correlate our results to the SS-GRA model. As shown, the sequences derived by the two methods are nearly identical. Their respective strengths, however, were the major point of distinction. Although the GRA model dimensions and Pearson's correlational coefficient are not identical, their judgments may be contrasted since both scales allow the decision-maker to assess if the association is moderate, significant, or extremely powerful.

### The Hurwicz's (Min-Max) approach

The Hurwicz (Min-Max) metrics have been referred to as the realism criterion. This method is used to make judgments (decisions) in conditions of uncertainty. It is commonly used in decision-making when we are challenged with several alternatives and an uncertain natural context. This procedure followed the stages outlined by Sikdar (48). We need to reduce cardiac DALYs for the current study, hence the decision would be as follows:


$$\begin{array}{l} \min A_{k}\left\{\alpha \;\max\;N_{p}\;v\;\left(A_{k},N_{p}\right)+\left(1-\alpha \right)\;min\;N_{p}\;v\;\left(A_{k},N_{p}\right)\right\} \end{array}$$

The metric is classified as an optimism index, and its magnitude ranges from [0–1]. In this case, we'll use 0.8. The following are the outcomes of a minimization approach:

A_1_ (IHD): (0.8 × 0.9912) + (0.2 × 0.7007) = 0.9331
A_2_ (Stroke): (0.8 × 0.9876) + (0.2 × 0.7202) = 0.9341

The results indicated that the prevalence of IHD is more likely to be affected by the risk factors attributable to nutrition in the Spanish population as compared to stroke. As an aggregate, the projected outputs of both decision-making approaches demonstrated that low-vegetable-and-fruits are strongly related to an upsurge in overall cardiac prevalence, specially IHD.

## Discussion

Health professionals and policymakers are tremendously concerned about the fact that CVDs are the leading cause of morbidity and death in Spain. Chronic illnesses, particularly CVD, are greatly influenced by poor nutrition. To reduce the prevalence and mortality from CVDs, much research has been done on the role of dietary variables in morbidity linked with cardiovascular disorders. However, our research takes a step further by assessing the degree of association and effect between the selected variables (nutritional risk factors) and morbidity spurred on CVDs, particularly IHD and stroke, in the Spanish population from 2010 to 2019. In the current investigation, we implemented three grey relational models (Deng GRA, absolute GRA, and SS-GRA models), which might potentially replace conventional data analysis techniques. The estimated statistics confirmed the suitability and effectiveness of the grey relational model technique.

The findings exhibited that, among the variables chosen, a diet poor in fruits tended to be highly grounded when compared with stroke morbidity in Spain, but a diet low in vegetables is the most likely contributor to the occurrence of IHD. Contrarily, in our analyses, zinc and iron insufficiency were the factors that had the least impact on morbidity from IHD and stroke. Additionally, we contrasted the outcomes of our advanced mathematical grey modeling analysis with those of a more conventional statistical technique, i.e., Pearson's correlation. The ranking order derived from the grey technique and that derived from the correlation was found to be identical, confirming the reliability of the grey analyses. Moreover, leveraging the statistics from the SS-GRA, a decision analysis technique (Hurwicz's criterion) was also applied to verify the robustness of the results. This approach confirmed a strong relationship between nutritional variables and the prevalence of IHD. Interestingly, we also examined a strong relationship between IHD and stroke morbidity caused by nutritional risk factors, which highlights nutritional factors as a potential contributor to improving heart health among the Spanish population.

Prior research has established a nexus between a diet poor in fruits and vegetables and the development of heart diseases, particularly IHD and stroke, in patients (49, 50). The best dietary practices for cardiac protection receive less attention than other dietary factors that may affect IHD and stroke risk. The findings of our study make it abundantly evident that a diet low in fruits and vegetables has a significant impact on the onset and progression of IHD and stroke, and they emphasize that one of the best dietary patterns for prevention is to increase the consumption of fruits and vegetables. It is challenging to specify an individual food component a pathophysiological function due to the great diversity of nutritional research. There may be intricate relationships between the various diet components, and any influences cannot be attributable to a single diet component but rather to a conjunction of dietary elements (51). Our results are aligned with the previous investigations which have been conducted to investigate the role of diet in cardiac health (52–56). An abundance of observational and experimental data suggests that practicing the Mediterranean diet (MD) is strongly correlated with a reduced risk of cardiac-related disorders (57). In particular, no other dietary pattern has accumulated as much data as the MD. Numerous observational analyses have explored the association between mortality and the incidence of MD and CVD (58–60). Several meta-analyses that compiled their risk assessments revealed an overarching inverse correlation (61–64).

In the going era, it has become more challenging to maintain heart-healthy dietary behaviors since the food environment has a considerable impact on people's food preferences, nutrition quality, and ergo cardiac health in several aspects. There are several systematic federal, state, and municipal activities and regulations that hinder the acquisition of these dietary behaviors against the backdrop of widespread nutrition ignorance. In this scientific claim, aspects such as specific food advertising, compositional racial prejudice, neighborhood discrimination, unpleasant built environmental conditions, and dietary and nutritional uncertainty all contribute to environments where unhealthy diets are predefined choices where we ingest, work, and reside. Furthermore, differences in product access, affordability, pricing, advertising, and placement in various locations sometimes make it convenient to consume harmful food as compared to healthier options. Improving food and nutrition quality, as well as chronic health issues across all communities will necessitate resolving these underlying structural issues, especially among persons of marginalized races and nationalities (2, 65). One significant adjuvant technique is to vigorously combat dietary myths among the general public and medical professionals, in conjunction with significant environmental improvements. Such endeavors may be aided by the inclusion of food and nutrition awareness in elementary and medical school curricula (66, 67).

The National Institutes of Health (NIH) (2020–2030 Strategic Plan for Nutrition Research) emphasizes precision nutrition to identify the influence on the health of not just what people consume, but also when, why, and how they consume throughout their lives (68). Precision nutrition is based on mounting data on those individual disparities in food consumption, habits, genetic history, microbial, and demographic and physical settings that impact disease susceptibility. The proactive goal of the NIH intends to deepen awareness of the synergies among these determinants to enable the establishment of significant clinical treatments to optimize food ingestion and the wellness of individuals. Precision nutrition integrates bioinformatics, genomics, and artificial intelligence with operational and cognitive disciplines (68–70). Multiplex precision nutrition techniques may significantly eradicate demographic, racial, and cultural differences in food consumption and CVD outcomes in the coming decades (71). Nonetheless, while precision nutrition can deliver tailored diets for cardiac prevention in the future, the discipline is still in its early stages. As a result, the present emphasis on public health nutrition endeavors to strengthen the food environment is substantial.

Since it facilitates clarity, resilience, and consistency in the presence of numerous and conflicting criteria, scientists have concluded that the MCDA paradigms are efficacious in the public health care contexts and are a viable decision-making technique [12, 45]. The findings of the present study suggest that when challenged with several options of comparable significance in healthcare decision-making scenarios, experts should leverage MCDA approaches and tools. The results of this study could assist researchers with mixed-method research guidance to aid in improving the excellence of their works and their comprehension of how to employ multi-method approaches to assess and prioritize the contributing variables of disease fatality in healthcare analytics. Such studies may aid in enhancing our ability to get key perspectives into the complex structure of the variables in a framework. Moreover, the recommended techniques also offer policy and decision-makers a useful tool and more in-depth practical information to aid in generating meaningful conclusions.

## Conclusion

In conclusion, a balanced diet, particularly fruits and vegetables, has a direct influence on IHD and stroke disorders and lowers the risk of cardiac events. This investigation strongly supports a balanced diet and precision nutrition guidelines as a strategy for reducing cardiac-related diseases in the Spanish population. It is a public health primary consideration to build an ambiance that encourages, rather than hinders, compliance with cardioprotective dietary practices among all people.

## Data availability statement

Publicly available datasets were analyzed in this study. This data can be found here: https://www.healthdata.org/gbd/2019.

## Author contributions

Both authors listed have made a substantial, direct, and intellectual contribution to the work and approved it for publication.

## Funding

This work was supported by the Union Program of Science and Health of Hunan Province, China (2019JJ80011).

## Conflict of interest

The authors declare that the research was conducted in the absence of any commercial or financial relationships that could be construed as a potential conflict of interest.

## Publisher's note

All claims expressed in this article are solely those of the authors and do not necessarily represent those of their affiliated organizations, or those of the publisher, the editors and the reviewers. Any product that may be evaluated in this article, or claim that may be made by its manufacturer, is not guaranteed or endorsed by the publisher.
